# The many hats of transmembrane emp24 domain protein TMED9 in secretory pathway homeostasis

**DOI:** 10.3389/fcell.2022.1096899

**Published:** 2023-01-16

**Authors:** Benjamin S. Roberts, Prasanna Satpute-Krishnan

**Affiliations:** Department of Biochemistry and Molecular Biology, Uniformed Services University of the Health Sciences, Bethesda, MD, United States

**Keywords:** transmembrane emp24 domain, p24 family, cargo receptor, autophagy, secretory pathway homeostasis, COP Coatomer, endoplasmic reticulum, Golgi

## Abstract

The secretory pathway is an intracellular highway for the vesicular transport of newly synthesized proteins that spans the endoplasmic reticulum (ER), Golgi, lysosomes and the cell surface. A variety of cargo receptors, chaperones, and quality control proteins maintain the smooth flow of cargo along this route. Among these is vesicular transport protein TMED9, which belongs to the p24/transmembrane emp24 domain (TMED) family of proteins, and is expressed across vertebrate species. The TMED family is comprised of structurally-related type I transmembrane proteins with a luminal N-terminal Golgi-dynamics domain, a luminal coiled-coil domain, a transmembrane domain and a short cytosolic C-terminal tail that binds COPI and COPII coat proteins. TMED9, like other members of the TMED family, was first identified as an abundant constituent of the COPI and COPII coated vesicles that mediate traffic between the ER and the Golgi. TMED9 is typically purified in hetero-oligomers together with TMED family members, suggesting that it may function as part of a complex. Recently, TMED family members have been discovered to play various roles in secretory pathway homeostasis including secreted protein processing, quality control and degradation of misfolded proteins, and post-Golgi trafficking. In particular, TMED9 has been implicated in autophagy, lysosomal sorting, viral replication and cancer, which we will discuss in this Mini-Review.

## Introduction

The first member of the transmembrane emp24 domain (TMED) family proteins, TMED11, was discovered in rough microsomes derived from canine endoplasmic reticulum (ER) in 1991 (Wada et al., 1991). Within a few years, TMED9 and other TMED family proteins were found to be type I transmembrane COPI and COPII coatomer binding proteins localized to the secretory pathway and conserved across mammals, yeast, and plants (Schimmoller et al., 1995; Stamnes et al., 1995; Belden and Barlowe, 1996; Elrod-Erickson and Kaiser, 1996; Sohn et al., 1996; Dominguez et al., 1998; Contreras et al., 2004a; Contreras et al., 2004b). The TMED family was originally referred to as the p24 family after their size (∼24 kDa), subfamily (ɑ, β, δ, or γ), and the order in which they were identified (1–5) (Strating et al., 2009). Each TMED protein has several aliases. Strating et al. (2009) organized the names in a useful reference table.

The secretory pathway is the major biosynthetic hub for the production, secretion, and turnover of soluble secretory and transmembrane proteins in eukaryotic cells. Traffic through the secretory pathway begins at the ER, where proteins are synthesized, folded, and processed prior to export to the Golgi for subsequent transport to the cell surface or lysosomes. Within the early secretory pathway, which is comprised of the ER, ER-Golgi intermediate compartment (ERGIC) and Golgi, protein folding is aided and monitored by chaperones and protein quality control (PQC) machinery (Anelli and Sitia, 2008; Adams et al., 2019; Sun and Brodsky, 2019). While TMED9 and TMED family members are well-characterized as regulators of homeostasis and vesicular transport within the early secretory pathway (Strating and Martens, 2009; Pastor-Cantizano et al., 2016; D'Arcangelo et al., 2015; Belden and Barlowe, 2001; Elrod-Erickson and Kaiser, 1996), their precise functions within this area are yet to be determined.

TMED family proteins promote efficient and selective secretion of diverse classes of proteins. TMED family members, including TMED10 and TMED2, facilitate ER-export of glycosylphosphatidylinositol-anchored proteins (GPI-APs) in yeast and cultured mammalian cells (Muniz et al., 2000; Marzioch et al., 1999; D'Arcangelo et al., 2015; Schimmoller et al., 1995; Belden and Barlowe, 2001; Fujita et al., 2011), and are required for ER-export of misfolded GPI-APs destined for lysosomal degradation (Satpute-Krishnan et al., 2014; Sikorska et al., 2016; Zavodszky and Hegde, 2019). TMED10 was recently shown to promote the unconventional protein secretion (UPS) of leaderless cargo including mature IL-1β (Zhang M. et al., 2020). TMED9 in particular has emerged as a major regulator of secretory pathway protein homeostasis through its involvement in protein trafficking and degradation. TMED9 has a propensity to form and function as a hetero-oligomer with other TMED family members (Belden and Barlowe, 1996; Fullekrug et al., 1999; Muniz et al., 2000; Fujita et al., 2011). Therefore, in this review we will describe TMED9 in the context of the larger TMED family of proteins.

## The TMED family of secretory pathway proteins

### TMED protein expression patterns

In humans there are 11 genes annotated as TMED1-11. TMED family members are expressed throughout the body, as demonstrated in mice (Strating et al., 2009), and are highly expressed in secretory cell types (Zhang and Volchuk, 2010). Given their ubiquity, it is thus unsurprising that some TMED proteins are developmentally essential and knockout of either *TMED2* or *TMED10* is embryonic lethal in mice (Denzel et al., 2000; Jerome-Majewska et al., 2010) and reduces viability in cultured cells (Blomen et al., 2015).

Various studies have shown that the TMED proteins form oligomers of varying stoichiometry (Fullekrug et al., 1999; Emery et al., 2000; Jenne et al., 2002). A series of siRNA knockdown experiments revealed that knockdown of TMEDs 2, 4, 5, 9, or 10 destabilized other TMED family members while TMED7 knockdown primarily affected TMED5. Loss of TMEDs nine or 10 inhibited GPI-AP trafficking, whereas WNT trafficking was inhibited in cells lacking either TMEDs 2, 4, 9, or 10 (Tashima et al., 2022). Because of the interdependency between TMED family member expression and function, it is technically challenging to discriminate between the functions of individual TMED proteins or their oligomeric complexes.

### The role of TMED9 in the secretory pathway

Mammalian TMED9 and its yeast homolog, Erv25p, were first discovered as secretory pathway proteins (Dominguez et al., 1998; Marzioch et al., 1999). TMED9 localizes primarily to the ER and ERGIC, but is found in post-Golgi secretory vesicles along with other TMED family proteins (Shevchenko et al., 1997; Dominguez et al., 1998; Marzioch et al., 1999; Breuza et al., 2004). Later TMED9 was discovered to be critical for the generation of ER exit sites (ERES) in a cell-free microsome budding assay (Lavoie et al., 1999). Further emphasizing its role(s) in the secretory pathway, depletion of TMED9 leads to the fragmentation of Golgi structures and the partial dissociation of COPI from the Golgi (Mitrovic et al., 2008). The yeast homolog of TMED9, Erv25p, has been shown to be play a role in efficient ER-to-Golgi transport of the yeast GPI-AP, Gas1 (Belden and Barlowe, 1996). However teasing apart TMED9’s individual role from other TMED-family members, including TMEDs 2 and 10, is difficult because knockdown of each impacts the expression of the others (Fujita et al., 2011). Taken together, TMED9 along with its family appears to regulate multiple critical trafficking steps in the secretory pathway. Excellent reviews have been written to discuss the role of the TMED proteins in the early secretory pathway (Pastor-Cantizano et al., 2016; Aber et al., 2019).

Recently, TMED9 was shown to participate in unconventional protein secretion (UPS) from the ER to the plasma membrane during ER stress in cells expressing the dominant-inhibitory form of ADP-ribosylation factor 1 (ARF1-Q71L), which blocks ER-to-Golgi transport (Park et al., 2022). TMED9 was found to participate in the assembly of a heterooligomeric trafficking complex governing SARS-Cov2 spike protein and cystic fibrosis transmembrane conductance regulator (CFTR) secretion (Park et al., 2022). Although Park et al. (2022) found that TMED9 did not bind to CFTR or Spike proteins, silencing TMED9 reduced the cell surface trafficking of these UPS cargo. These findings suggest that TMED9 may participate in a variety of yet undiscovered trafficking pathways.

### Structure-function relationships in the TMED family

**FIGURE 1:**
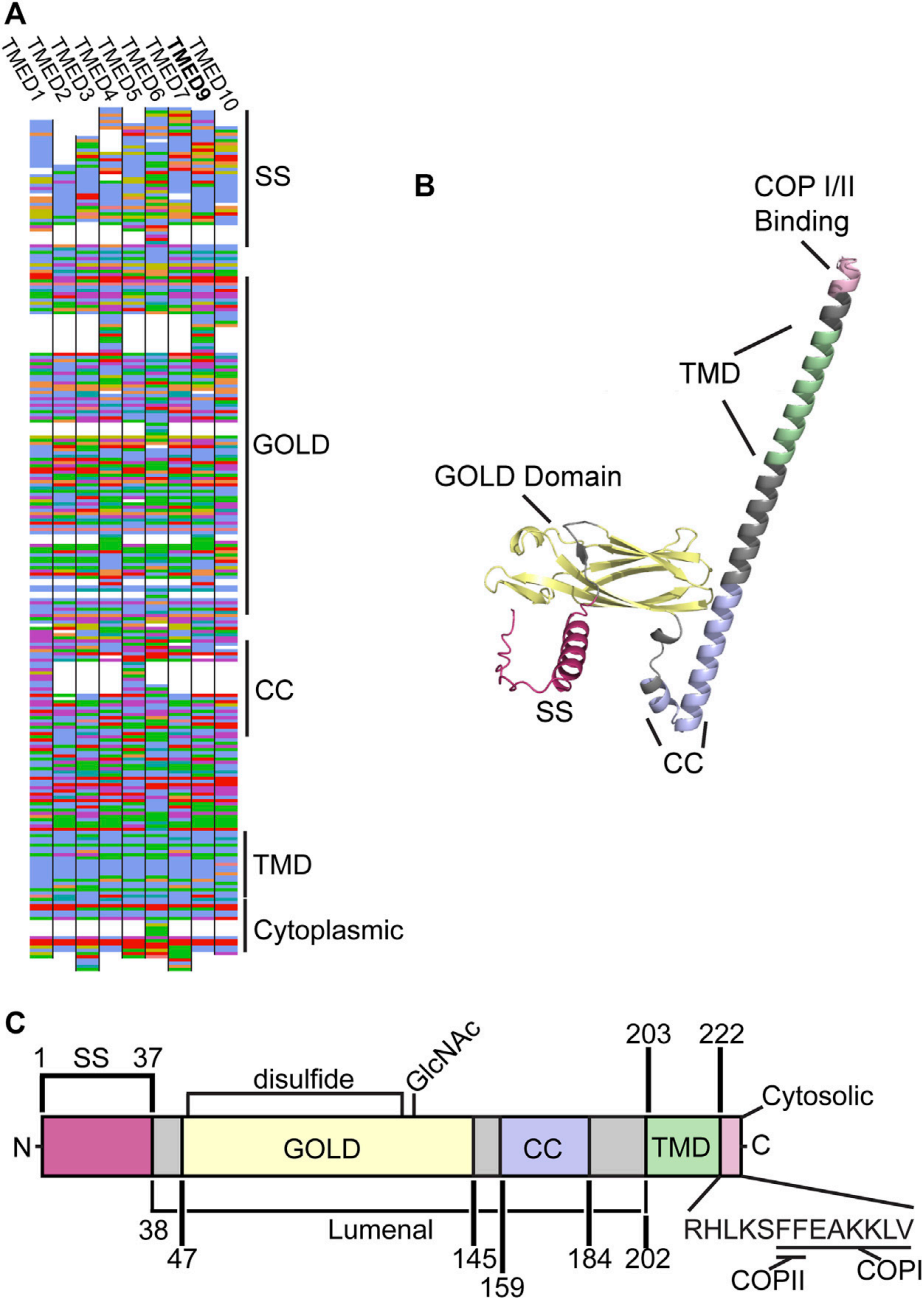
**(A)** The protein sequence for human TMEDs 1, 2, 3, 4, 5, 6, 7, 9, and 10 are shown. Sequences were aligned with Muscle and drawn with AlignmentViewer (alignmentviewer.org). Aligned amino acids are colored in the Clustal2 color code. Structural motifs for TMED9 are indicated (SS: signal sequence, GOLD, CC: coiled-coil, TMD: transmembrane domain, COP: COPI/II). **(B)** The Alphafold (Jumper et al., 2021; Varadi et al., 2022) structure for the human TMED9 protein (AF-Q9BVK6-F1). The signal sequence (SS, red), GOLD domain (yellow), coiled-coil (CC, lilac), transmembrane domain (TMD, green), and COP I/II (pink) binding sites are indicated **(C)** A predicted domain map of the human TMED9 protein (Q9BVK6) as compiled and annotated by Uniprot (UniProt, 2021). The structural domains from the N-terminus “N” to the C-terminus “C” in **(B)** are indicated, as well as a conserved disulfide bond, N-linked glycan (GlcNAc), and the COPI and COPII binding sites. Amino acid positions are given and domains are depicted to scale.

The TMED proteins are structurally conserved among eukaryotes despite significant variations in sequence identity (Strating et al., 2009) (Figure 1A). Each family member contains four major regions: the GOLD domain, coiled-coil domain, transmembrane domain, and a cytoplasmic COP-binding region (Figures 1B, C). Whether these conserved domains allow the TMED proteins to act interchangeably in certain processes is unknown.

#### GOLD domain

The Golgi dynamics (GOLD) domain consists of eight β-strands and one disulfide bond (Nagae et al., 2016). Despite low sequence homology, the GOLD domains found in TMEDs 1, 2, 5, and 10 are structurally similar (Nagae et al., 2016; Nagae et al., 2017; Mota et al., 2022). The GOLD domain is chiefly involved in hetero and homo-oligomerization (Nagae et al., 2016; Zhang M. et al., 2020; Mota et al., 2022). Heterodimerization occurs across a range of sites on each GOLD domain, depending on the TMED proteins involved (Nagae et al., 2016). Dimerization is dependent on solution ionic strength (Mota et al., 2022) and pH (Nagae et al., 2016) *in vitro*, suggesting that intracellular localization may influence dimerization. These findings are largely sourced from studies involving purified GOLD domains rather than intact TMED proteins. Beyond its role in oligomerization, the GOLD domain has also been shown to participate in substrate recognition (Park et al., 2022) and the interaction between TMEDs 9 and 10 with syntaxin 17 (Muppirala et al., 2011).

#### Coiled-coil domain

The coiled-coil (CC) domain mediates TMED oligomerization and substrate recognition. Early observations showed that CC deletion abolished post-ER transport of hetero-oligomeric complexes (Ciufo and Boyd, 2000; Emery et al., 2000) and recently the TMED7 CC was shown to participate in TMED7 homooligomerization (Liaunardy-Jopeace et al., 2014). Recent studies have also shown that the CC domain appears to mediate substrate recognition in the case of GPI-anchored proteins (Theiler et al., 2014) and TLR4 complex binding (Liaunardy-Jopeace et al., 2014).

#### Transmembrane domain

The transmembrane domain (TMD) is essential in TMED protein sorting. The TMD of TMED2 but not TMED10 binds to sphingomyelin (SM) C18, promoting TMED2 dimerization and regulating cargo transport (Brugger et al., 2000; Contreras et al., 2012; Aisenbrey et al., 2019; Pannwitt et al., 2019). It is unclear if other TMED family members interact with SM in this way. Because membrane lipid content can affect membrane thickness, TMD length and lipid binding may increase the affinity of TMED proteins for membrane microdomains that are enriched with SM. Whereas no defined sorting motif has been identified within TMED protein TMDs, lengthening the TMED10 TMD impacts that protein’s sorting (Blum and Lepier, 2008). Intriguingly, it has been reported that membrane SM content affects the formation of coatomer protein (COP)-marked vesicles (Brugger et al., 2000). It is possible this effect is mediated by TMED proteins, since they interact with membrane lipids and COP (Pannwitt et al., 2019).

#### Cytoplasmic domain

The cytoplasmic tail of the TMED proteins contains a region required for COP I/II binding, which we will discuss below. In addition, the cytoplasmic domain has been shown to bind to mature IL1β for TMED10 (Zhang M. et al., 2020), and syntaxin 17 and TC48 for TMEDs 9 and 10 (Muppirala et al., 2011; Muppirala et al., 2013). Intriguingly, the TMED9 cytoplasmic domain was also recently implicated in the formation of autophagic vesicles through its interaction with Sec12, the guanine-nucleotide exchange factor for Sar1 that functions upstream of COPII coat assembly (Weissman et al., 2001; Li et al., 2022).

COPI proteins bind to dilysine (KKXX) motifs and structurally related sites in cargo proteins (Ma and Goldberg, 2013). Of the TMEDs, only TMEDs 4, 9, 10, and 11 include a canonical KKXX motif, however KXK motifs in some TMED orthologs also enable COPI binding (Teasdale and Jackson, 1996; Dominguez et al., 1998; Belden and Barlowe, 2001; Pastor-Cantizano et al., 2016; UniProt, 2021). Consequently, TMEDs 9 and 10 have been shown to bind COPI components more strongly than TMEDs 2, 3, or 7. COPI binding is important for TMED retrieval from the Golgi back to the ER (Bremser et al., 1999) and mutations of this motif in TMEDs 2, 9, and 10 alters their ER-Golgi cycling kinetics (Dominguez et al., 1998; Blum and Lepier, 2008). Interestingly, COPI components recognize TMED oligomers rather than TMED monomers (Bethune et al., 2006).

COPII binding to the TMED proteins is mediated through aromatic residues in the cytoplasmic domain which fit into a binding pocket in the SEC24 COPII coat proteins (Ma et al., 2017). While all TMED family proteins display cytoplasmic aromatic residues, variations in the polypeptide sequence enable different TMED proteins to associate with different SEC24 isoforms (Wendeler et al., 2007).

## TMED9 interactions in health and disease

Only a handful of diseases have been directly linked to TMED9. However, TMED family proteins have been tied to a variety of human diseases. Because the TMED proteins function as heteromeric complexes, TMED9 is likely to participate in some of the diseases associated with other TMED family members. Thus, we have listed diseases associated with each of the TMED proteins in Table 1.

**TABLE 1:** Diseases associated with individual TMED family proteins.

Protein	Associated diseases	Additional reference
TMED1	Cardiovascular disease	Liew et al. (2010); Connolly et al. (2013)
TMED2	Non-alcoholic fatty liver disease	Hou et al. (2017)
TMED3	Colon cancer	Duquet et al. (2014)
TMED4		
TMED5	Cervical cancer	(Yang et al., 2019; Yang et al., 2021)
TMED6	Diabetes	Wang et al. (2012)
TMED7	Amyotrophic lateral sclerosis	(Pradat et al., 2012)
TMED9	Breast cancer, Colon cancer, Head and neck squamous cell carcinoma, Hepatocellular carcinoma, Mucin-1 kidney disease, Epithelial ovarian cancer	Dvela-Levitt et al. (2019); Mishra et al. (2019); Ju et al. (2021); Yang et al. (2021); Han et al. (2022)
TMED10	Alzheimer’s disease	Chen et al. (2006); Shin et al. (2019)

### Cancer

Elevated *TMED9* expression has been observed in multiple cancer types (Ju et al., 2021). In breast cancer, elevated TMED9 levels are associated with poor prognoses (Ju et al., 2021). In head and neck squamous cell carcinoma, expression of each of the TMED proteins is elevated. In particular, high expression of *TMEDs 2, 9,* and *10* was found to be associated with poor prognoses, whereas high expression of TMEDs 1, 3, 4, 5, 6, and 7 was not (Gao et al., 2022). Similarly, elevated *TMED9* expression is associated with reduced survival time in individuals with epithelial ovarian cancer (EOC) *in vivo.* TMED9 knockdown reduces EOC cell proliferation *in vitro* (Han et al., 2022).

TMED9 expression may regulate cancer cell proliferation through its effect on growth factor signaling (Buechling et al., 2011; Nakano et al., 2017; Zhang X. et al., 2020; Di Minin et al., 2022; Tashima et al., 2022). For example, biochemical and microscopy approaches revealed that TMED9 loss was associated with dysregulation of TGFɑ trafficking and secretion in colon cancer and hepatocellular carcinoma cells (Mishra et al., 2019; Yang et al., 2021). Furthermore, loss of TMED9 led to impaired WNT trafficking (Tashima et al., 2022) and significant changes in the expression of genes regulated by WNT signaling (Yang et al., 2021). This TMED9-WNT signaling axis has been implicated in Paneth cell function in the intestines (Goga et al., 2021).

### Neurodegenerative disease

The TMED proteins have been implicated in various neurodegenerative diseases. TMED10 associates with and is required for the clearance of artificial and prion-disease associated mutants of prion protein (PrP) (Satpute-Krishnan et al., 2014). TMED2 was found to co-immunoprecipitate with atlastin whose misfolding leads to hereditary spastic paraplegia (Namekawa et al., 2007). Although TMED9 has not been thoroughly studied in the context of neurological disease, TMED9 has been shown to interact with wild type TDP-43, whose aggregation has been associated with the development of amyotrophic lateral sclerosis (ALS) (Redler and Dokholyan, 2012; Feneberg et al., 2020), and to associate with spastin whose mutations lead to hereditary spastic paraplegia (Reid et al., 2005). The precise motifs or domains of TMED9 involved in binding to TDP-43 or spastin remain unknown.

Various studies have demonstrated that alterations in TMED9 and TMED10 (commonly referred to as TMP21 in the Alzheimer’s field) expression promote the processing of amyloid precursor protein (APP) to amyloid beta (Aβ) by γ-secretase. Alzheimer’s disease has been associated with mutations in the genes encoding subunits of γ-secretase (Zhang et al., 2011). A single nucleotide polymorphism in *TMED10* that resulted in heightened TMP21 expression was found to be genetically associated with Alzheimer’s disease in patients (Zhang et al., 2018). Additionally, alterations in *TMED10* expression were found to impact pathological APP processing in cell culture models (Zhang et al., 2018). TMED10/TMP21 has been shown to co-immunoprecipitate with and regulate the activity of the γ-secretase complex. Intriguingly, depletion of TMED10/TMP21, results in increased generation of Aβ (Chen et al., 2006; Dolcini et al., 2008). Similarly, TMED9 co-immunoprecipitates with the core γ-secretase components and knockdown of TMED9 mRNA induces an increase in Aβ generation (Hasegawa et al., 2010; Bai et al., 2015). Because of TMED9’s tendency to heterooligomerize with TMED10/TMP21 (Dominguez et al., 1998; Fullekrug et al., 1999), TMED9 may function in a complex with TMED10/TMP21 to regulate γ-secretase processing of APP.

### Mucin kidney disease

The proteinopathy mucin-1 kidney disease (MKD) results from a frameshift mutation in the *MUC1* gene. (Dvela-Levitt et al. (2019) recently demonstrated that TMED9 binds to and mediates the post-ER trafficking of MUC1 aggregates. Under steady-state conditions, this TMED9-MUC1 complex drives the accumulation of toxic MUC1 in the ERGIC. Fortuitously, the authors found that the small molecule BRD4780 was able to reduce MUC1 aggregate levels both *in vivo* in mice and *in vitro* in cell culture and human kidney organoid models by reducing TMED9 stability and accelerating clearance of TMED9-MUC1 complexes from the ER and ERGIC to lysosomes (Dvela-Levitt et al., 2019).

## TMED9 in autophagy

Over the last decade, TMED9 has emerged as an important regulator of cellular proteostasis. It has recently been shown that TMED9 contributes to autophagy and autophagosome biogenesis. TMED9 was first identified in intracellular vesicles enriched with ATG9 and Rab1 thought to participate in autophagosome assembly (Kakuta et al., 2017). A role for TMED9 in autophagosome maturation was later demonstrated by Evans et al. (2021). The authors found that TMED9 knockdown attenuated autophagic activity and reduced viral production, potentially by decreasing COPII-dependent viral transport (Delorme-Axford et al., 2014; Evans et al., 2021).

In line with these findings, TMED9 was recently shown to directly participate in autophagosome biogenesis. It has long been known that TMED9 participates in ER exit site (ERES) formation for cargo transport to the Golgi (Lavoie et al., 1999; Fujita et al., 2011). However, Li et al. (2022) found that ERES-localized Sec12 and ERGIC-localized TMED9 interact directly in trans through their cytoplasmic domains, bringing ERES into close proximity with the ERGIC. ERES-ERGIC association is important for the generation of starvation-induced autophagosomes (Li et al., 2022). These findings suggest that TMED9 may directly influence the recruitment of COPII machinery at the ERGIC to contribute membranes for autophagosome formation.

## Discussion

TMED9 has been found in every organelle along the secretory pathway from the ER and Golgi, to the plasma membrane, to lysosomes and autophagosomes (Hasegawa et al., 2010; Li et al., 2022). While precise mechanistic functions of TMED9 remain elusive at each point, it is clear that TMED9 wears many hats in secretory pathway homeostasis. Building upon early findings that TMED9 binds to COPI and COPII coat proteins (Lavoie et al., 1999), recent studies indicate that TMED9 regulates the initial recruitment of COP machinery to the ERGIC membrane to promote the formation of autophagic membranes in coordination with COPII machinery (Kakuta et al., 2017; Evans et al., 2021; Li et al., 2022). Furthermore, *TMED9* expression correlates with the development of multiple cancer types. Roles for TMED9 in the regulation of cancer cell growth (Mishra et al., 2019; Ju et al., 2021; Yang et al., 2021), APP processing (Hasegawa et al., 2010; Bai et al., 2015), and protein degradation (Dvela-Levitt et al., 2019) underscore the importance of this cargo receptor in health and disease.

### Future directions

The TMED protein family field is a rapidly evolving area of research. The exciting discovery that BRD4780 targets pathological TMED9-MUC1 aggregates to lysosomes demonstrates the potential to pharmacologically target TMED proteins for the resolution of proteinopathies (Dvela-Levitt et al., 2019). It is as yet unclear how BRD4780 induces lysosomal degradation of TMED9-MUC1, but possible mechanisms may involve directly altering TMED9’s structure or by preventing its oligomerization with other TMED family members. Preventing hetero-oligomerization of TMED9 has been shown to reduce its stability and the stability of other TMED proteins (Tashima et al., 2022). Because of this interdependence, BRD4780 may potentially be exploited to modulate various PQC pathways involving TMED heterooligomers. These findings encourage future research into the role of TMED9 and the other TMED proteins in clinical proteinopathies.

Beyond their clinical implications, the TMED proteins have now been shown to participate in a variety of essential cellular processes. New empirical tools such as cryoelectron microscopy and AI based modeling tools like Alphafold2 enable future structural studies to better characterize the interaction of the TMED proteins with one another and their cargo. Furthermore, structure-driven mutagenesis strategies may be used to disrupt oligomer formation in order to reveal the independent functions of the TMED proteins in the secretory pathway. Compelling questions include: Does environmental pH affect the function and binding of TMED proteins? Does N-linked glycosylation influence TMED cargo recognition? What is the precise role of TMED9 in autophagosome biogenesis, and how is this balanced with its role in secretion? What are the specific roles of each TMED family member and their various oligomeric states? As we gain answers, we may soon understand the functions and clinical relevance of TMED9 and its stubbornly mysterious family of proteins.
